# Signs of current suicidality in men: A systematic review

**DOI:** 10.1371/journal.pone.0174675

**Published:** 2017-03-29

**Authors:** Tara Hunt, Coralie J. Wilson, Peter Caputi, Alan Woodward, Ian Wilson

**Affiliations:** 1 School of Psychology, University of Wollongong, Wollongong, Australia; 2 Illawarra Health and Medical Research Institute, Wollongong, Australia; 3 School of Medicine, University of Wollongong, Wollongong, Australia; 4 Lifeline Research Foundation, Lifeline Australia, Canberra, Australia; 5 Suicide Prevention Australia, Sydney, Australia; 6 Centre for Mental Health, University of Melbourne, Melbourne, Australia; University of Vienna, AUSTRIA

## Abstract

Suicide signs have been identified by expert consensus and are relied on by service providers, community helpers’ and family members to identify suicidal men. Whether signs that are reported in suicide literature accurately describe male presentations of suicidality is unclear. A systematic review of the literature was conducted to identify male-specific signs of current suicidality and identify gaps in the literature for future research. Searches through Medline, CINAHL, PsychInfo and the Behavioral Sciences Collection, guided by the PRISMA-P statement, identified 12 studies that met the study eligibility criteria. Although the results generally reflected suicide signs identified by expert consensus, there is little research that has examined male-specific signs of the current suicidal state. This review highlights the need for scientific research to clarify male presentation of suicidality. Implications for future research to improve the prompt identification of suicidal men are discussed.

## Introduction

Suicide is a leading cause of death worldwide, with 8 females and 15 males per 100 000 taking their own life by suicide in 2012 [1]. In higher socio-economic countries, such as Australia, there is a pronounced gender disparity with men three times more likely to take their own lives than women [1,2]. Factors contributing to this gender disparity include: men having increased access to more lethal means than women [3]; traditional male gender roles [4]; and male socialisation, including the reluctance to seek help [5]. Given the significant hazards associated with not detecting those experiencing current suicidality, the assumptions underlying current suicide intervention strategies warrant examination.

Suicide literacy refers to one’s knowledge of signs, causes and treatment of suicidality [6]. Suicide signs refer to indications of current suicidality that are *observed* by or *reported* to another, and indicate risk for suicide within minutes, hours or days [7,8]. Identified by expert consensus, widely accepted suicide signs include hopelessness, anger, recklessness, feeling trapped, social withdrawal, agitation, and mood changes [8]. They are commonly used as a tool to educate front-line community workers, mental-health professionals, and telephone crisis line operators for identifying people with current suicidality [9]. However, emerging research has questioned the sensitivity and specificity of these signs to identify the presence of current suicidality [10]. By definition, suicide signs are risk factors that operate over short time frames and need to be strongly associated with death by suicide because of the very low base rated of short-term suicide mortality. Gunn III, Lester and McSwain [10] tested the ability of common signs of suicide, such as suicide ideation, substance abuse, purposelessness, anger, trapped, hopelessness, withdrawal, anxiety, recklessness and mood change, to differentiate between suicidal ideators, non-ideators and suicide attempters. Although all signs of suicide except substance abuse and anxiety differentiated ideators and non-ideators, only anger/aggression was predictive of suicide attempts. It is possible that the signs of suicide reported in current literature are insufficiently sensitive to accurately identify people experiencing current and immediate risk for death by suicide.

Identifying signs of suicide in men may be particularly important for reducing rates of male suicide. The expression and interpretation of mental health issues are significantly impacted by conceptions of gender and gender practices [11]. Men may discuss their distress in ways that are not immediately recognisable [12,13], resulting in male mental health problems being undetected or ‘hidden’ [14]. There is additional evidence that mental health issues such as depression [15,16], PTSD [17] and schizophrenia [18] have gender-specific signs. Research into suicide signs is yet to systematically examine the impact of gender. The purpose of this review is to identify male-specific signs of current suicidal risk that are reported in the suicide literature and gaps in the literature for future research. To ensure that suicidal men experiencing are recognised it is vital that helpers and community members have tools that are able to accurately identify signs indicative of suicidality.

Suicide signs may be different given type of suicidality [7]. This review investigates signs for three levels of suicidality—suicidal ideation, suicide attempt, and death by suicide—as separate categories of suicidality. Suicidal ideation refers to an individual thinking about intentional self-injury, where intent refers to the aim, purpose or goal of a behaviour [19], and there is evidence that the person intends at some undetermined or some known degree, to kill him or herself [20]. Suicide attempt refers to the situation where intentional self-injury is carried out. Death by suicide is defined as an individual taking his or her own life, and there is evidence that the person intended to kill themselves.

The current review seeks to identify male-specific signs of current suicidality by reviewing current evidence in the research literature, and identify gaps in the current literature about male suicidal presentation to provide directions for future research.

## Method

### Search strategy

This systematic review of the literature was guided by the Preferred Reporting Items for Systematic Reviews and Meta-Analysis Protocol (PRISMA-P) statement, which provides an evidence-based protocol for developing systematic reviews and meta-analyses [21]. Electronic databases (Medline, CINAHL, PsychInfo and Behavioral Sciences Collection) were systematically searched between November 2014 and August 2015 and refreshed in October 2016. These databases represent the broadest range of peer-reviewed literature across the disciplines of psychology, medicine and allied health. Search terms included ‘men’, ‘male’, ‘suicid*’, ‘self-harm*’, ‘self-injurious behavio*’, ‘warning sign’, ‘symptom’, ‘sign*’, ‘predict*’, ‘indicat*’ (see Table 1). The search was limited to research published from 2000 to 2015 because we were interested in understanding the latest research in assessing male-specific signs and most research before this time is referred to and built on in the articles that were selected.

**Table 1:** Search strategy by database.

Database	Search strategy
Medline	MW (("men" OR "male")) AND MW ((suicid* OR "self-harm*" OR "self-injurious behavio*)) AND TX (("warning sign" OR symptom OR sign* OR predict* OR indicat*)), Limiters: Full text, published 2000–2015, English language only, MW = Word in subject heading, TX = full text
CINAHL	MW (("men" OR "male")) AND MW ((suicid* OR "self-harm*" OR "self-injurious behavio*)) AND TX (("warning sign" OR symptom OR sign* OR predict* OR indicat*)), Limiters: Full text, published 2000–2015, English language only, MW = Word in subject heading, TX = full text
PsychInfo	1# (men or male).mp. [mp = title, abstract, heading word, table of contents, key concepts, original title, tests & measures]; 2# suicid*.mp.; 3# ("self-harm" or self-injurious behavio* or "suicide attempt").mp.; 4# 2 or 3; 5# "warning sign".mp.; 6# symptom.mp; 7# predict*.mp; 8# indicat*.mp; 9# sign*.mp.; 10# 5 or 6 or 7 or 8 or 9; 11# 1 and 4 and 1012# limit 11 to (full text and english language and yr = "2000 -Current")
Psychology and Behavioural Sciences Collection	AB (("men" OR "male")) AND AB ((suicid* OR "self-harm*" OR "self-injurious behavio* OR "suicide attempt")) AND TX (("warning sign" OR symptom OR sign* OR predict* OR indicat*)), Limiters: Full text, published 2000–2015

### Eligibility criteria

Studies selected for inclusion in the systematic review met the following criteria: (1) contained original research employing a quantitative or qualitative research design; (2) were conducted in a population aged between 18 and 65 years inclusively, as suicidal presentation in adolescents and older people includes population-specific features; (3) included a sample where participants experienced suicidal thoughts, suicidal plans, suicide attempts or deaths by suicide; (4) reported male and female, or male only suicide signs and suicidal ideation or behaviour separately; and (5) identified suicide signs within 4 weeks of suicidal ideation, suicide attempt, or death by suicide.

The timeframe is particularly important as research into suicide signs has often not taken timeframe into account [22]. Because of the difficulty associated with assessing signs of suicide in the minutes or hours prior to death [9], the ‘near term’ timeframe for this study was extended to 4 weeks. This timeframe was selected as widely used measures of suicidal ideation and behaviour, such as the Beck Depression Scale [23] and Hamilton Depression Rating Scale [24], hold 4 weeks as a cut-off point for current suicidal ideation or behaviour.

### Data extraction

After duplicates were removed, study titles were screened to obtain full text articles for review. Full-text articles were imported into a Microsoft Excel data file to manage the records throughout the review and selection process. Full-text articles were screened, and all articles that did not fulfil the eligibility criteria were excluded. The articles obtained in the review were then appraised by three of the authors to ensure eligibility for inclusion; any disagreements were resolved through discussion. An article summary sheet was developed to record data from each eligible study. Recorded data were: sample, setting, design, measurement of suicidal ideation or behaviour, signs of suicide in men, and relevant findings.

### Risk-of-bias

The 13 articles that fulfilled the criteria for inclusion in the systematic review underwent a three-step risk-of-bias (ROB) assessment to select final articles for analysis.

#### Quantitative ROB assessment

Quantitative studies meeting the inclusion criteria were assessed for ROB using the Effective Public Health Practice Project tool (EPHHP) [25]. The EPHPP is a ROB tool used across a wide variety of quantitative designs; it has good validity [25] and inter-rater reliability [26]. Author TH conducted the ROB assessment and discussed decisions with authors CW and PC. The ROB assessment was also conducted by an independent reviewer. Interrater agreement was 89%, and any differences were resolved through discussion to reach 100% agreement.

#### Qualitative ROB assessment

Qualitative studies that met the inclusion criteria were assessed for ROB using the Critical Appraisal Skills Programme (CASP) checklist for qualitative studies [27]. The CASP tool focuses on three criteria—rigour, credibility, and relevance, and is therefore able to accommodate the diversity of methodological approaches within the qualitative domain. Dixon-Woods et al [28] found the CASP checklist to be the most reliable of ROB tools evaluated in their review. Author TH conducted the ROB assessment and discussed decisions with authors CW and PC. The qualitative appraisal was also conducted by an independent reviewer. Interrater agreement was 80%, and any differences were resolved through discussion to reach 100% agreement.

#### Global ROB rating

A global rating system was developed to integrate the findings of both qualitative and quantitative review tools. This process enabled the ROB assessment to be tailored to the specific needs of the review, which was to examine the best available literature identifying suicide signs. Initially the ROB ratings produced by the EPHPP prioritised research conducted using randomised controlled trials (RCTs). However, the requirement for RCTs can be unethical in suicide research [29]. Consequently, research articles that used alternative designs to RCTs were not downgraded and articles were deemed to have an acceptable ROB rating if the design was described in a rigorous, replicable way that matched the research questions and hypotheses, and clearly reported the reliability and validity of measures and analysis strategy. Articles that did not fulfil these criteria were categorised as being of a lower quality than acceptable for the current study.

#### Data coding

Due to the heterogeneous nature of the studies identified, and the broad nature of the review, a narrative synthesis approach was adopted as the most suitable method for the review [30]. A preliminary synthesis was conducted to develop an initial description of the pattern of male-specific suicide signs associated with suicidal ideation, suicide attempt, and death by suicide. Common phrases or themes identified in the results of articles were used to generate codes (see S1 Table and S2 Table for more information). The codes were agreed on by at least two coders, and if there was disagreement, codes were discussed until consensus was achieved. Signs identified in the qualitative and quantitative articles were then coded and categorised against suicidal ideation, suicide attempt, or death by suicide for the main narrative synthesis.

## Results

### Search results

**Fig 1:**
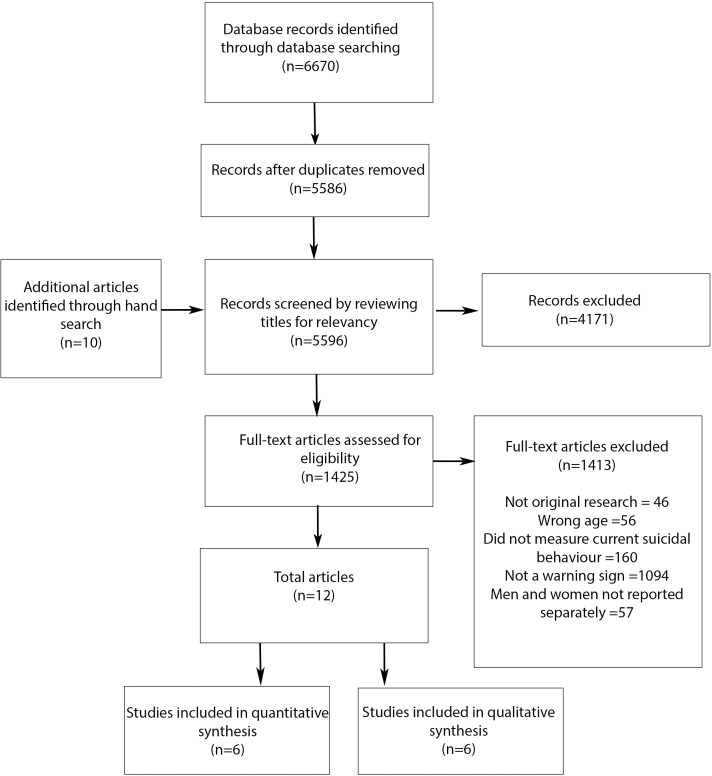
PRISMA-P flow chart of study selection.

The systematic literature review identified a total of 6770 articles (see Fig 1). After the removal of duplicates and title screening, 1425 articles were assessed for eligibility. Full-text articles assessed for eligibility were also hand searched for additional articles. A total of 12 studies were found to be eligible for inclusion in the narrative synthesis.

### Characteristics of identified studies

The 12 studies included a total of 8963 participants; 8394 participants had experienced some degree of suicidal ideation and/or behaviour, and 185 were informants for someone who died by suicide (Table 2).

**Table 2:** Studies included in synthesis.

First author (reference)	Study design, analysis method	Sample, gender, country (Number, % male, age)	Setting	Suicide category	Sign of current suicidality measurement	Sign of current suicidality label (see S1 Table for more detail)
Antypa (2013) [31]	Case-controlled design with three conditions (death by suicide, suicide attempt and control), MANOVA and MANCOVA	GER, suicide attempters (n = 171, 35.1% male, Age M = 52.11±18.45), suicide decedents (n = 90, 57.8% male), Age M = 39.87±13.95, Control (n = 317, 43.8% male, Age M = 45.05±14.87).	Inpatient sample, community sample and autopsy report.	Suicide attempt (psychiatric admission for attempt)/ suicide death (autopsy).	Self-report item (STAXI-2) [35]	• Anger
Bryan(2014) [36]	Cross-sectional design, multivariate linear regression	USA (N = 7698, 42.8% male, M = 39±18).	Inpatient sample.	Suicide attempt (self-report).	Self-report item (SSF-II-R) [37]	• Agitation
Eidhin (2002) [38]	Cross-sectional, ANOVA	IRL, Current ideators (n = 11, 100% male, age M = 21.9±4.8), past history of suicide (n = 12 100%, age M = 24.8±3.8), control group (n = 12, 100% male, age M = 25.6±10.7).	Prison inmate sample.	Suicidal ideation (self-report).	Self-report questionnaire (PPI& MEPS) [39,40]	• Paucity of problem solutions Social withdrawal
Humber (2013) [41]	Mixed-methods cross-sectional study, multi-level modelling	UK, (N = 21, 100% men, M = 36).	Prison inmate sample.	Suicidal ideation (self-report)	Ecological Momentary Assessment.	• Anger
Kiamanesh (2014) [42]	Psychological autopsy study, interpretive phenomenological analysis	NOR, suicide decendent sample (n = 6, 100% male, age M = 35.3±13.3), Informant sample (N = 41 59% male, age not specified).	Informants for suicide decedents.	Suicide death (autopsy)	Open-ended interviews.	• Irreversible defeat • Lack of sleep • Loneliness • Paucity of problem solutions
Lekka (2006) [43]	Case matched control with follow-up, Mann-Whitney U, Chi-square	GRC, Prisoners with suicidal ideation in past week (n = 67, 100% male, age M = 33.9±9.7), prisoners who have never experienced suicidal ideation, (n = 67, 100% male, age M = 33.7±9.7).	Prison inmate sample.	Suicidal ideation (self-report).	Self-report questionnaire.	• Excluded from study
Parker (2002) [32]	Psychological autopsy study, descriptive	AUS, Suicide decedents (N = 181, 82.3% male, age indigenous males M = 27.4; indigenous females M = 24.1; "other" males M = 37.7; "other" females M = 43).	Informants and autopsy report.	Suicide death (autopsy)	Open-ended interview.	• Excluded from study
Peters (2013) [44]	Qualitative narrative inquiry, content analysis	AUS, Suicide decedents (n = 10, 100% male, age = 20–60), Informant sample, (n = 10, 30% male, age = 25–65).	Informants on suicide decedents.	Suicide death (informant report)	Open-ended interview.	Direct statements of suicidal intent
Player (2015) [45]	Mixed method, thematic analysis	AUS, Men who attempted suicide 6–18 months prior (n = 35, 100% male, age Median = 43), family and friends of attempters (n = 47, 100% male, median age = 47).	Male suicide survivors and informants	Suicide attempt (self-report)	Open-ended interview.	• Aggression • Anger • Apathy • Calm • Direct statements of suicidal intent • Excessive risk taking • Hopelessness • Social isolation
Rasmussen (2014) [33]	Psychological autopsy study, interpretive phenomenological analysis	NOR, Suicide decedents (n = 10, 100% male, age = 18–30), informant sample (n = 61, % male not specified, age not specified).	Informants on suicide decedents.	Suicide death (autopsy)	Open-ended interview.	• Anger • Helplessness • Shame
Rasmussen (2014) [34]	Psychological autopsy study, interpretive phenomenological analysis	NOR, Suicide decedents (N = 10, 100% male, Age = 18–30), informant sample (n = 61, % male not specified, age not specified).	Informants on suicide decedents.	Suicide death (autopsy)	Open-ended interview.	• Agitation • Cheerfulness • Death as a problem solution • Desperation • Direct statements of suicidal intent • Emergence of positive change of mood • Indirect or ambiguous references to taking their own life • Paucity of problem solutions • Reaching out for support in desperation • Relief
Rivlin (2013) [46]	Semi-structured qualitative interview, thematic analysis	Male only sample, UK, Prison inmates (n = 60, 100% men, age not specified)	Prison inmate sample.	Suicide attempt (self-report)	Open-ended interview.	• Anger • Calm • Depression • Direct statements of suicidal intent • Feeling upset • Indirect or ambiguous references to taking their own life • Made arrangements for death • Planned suicide attempt • Relief • Wrote a suicide note

#### Sample of identified studies

The samples in the studies were obtained from a variety of contexts. The most frequently used strategy to identify eligible participants was through suicide registries and death records (n = 4, 33%)[31–34]. Samples were from prison inmate populations (n = 4, 33%), psychiatric wards (n = 2, 17%), self-selection from the wider population (n = 2, 17%). The sample sizes varied, ranging from 10 to 7698 participants. Most studies assessed suicide signs in men only (n = 9, 75%).

#### Design of identified studies

The sources of data were psychological autopsy studies (n = 4; 33%), cross-sectional survey designs (n = 2, 17%), case-controlled designs (n = 2; 17%), mixed method studies (n = 2; 17%), and studies using a mixture of qualitative designs (n = 2; 17%). Suicidal thoughts and behaviours were reported on men who had died by suicide (determined by coroner’s report) (n = 7, 50%), men who had been admitted to hospital for a suicide attempt (n = 2, 14%), men who self-reported they had attempted suicide (n = 1, 7%), and men experiencing suicidal ideation measured by self-report (n = 4, 25%). Suicide signs were predominantly identified by self-report measures (n = 3, 25%), informant reports (n = 6, 50%), or interviews with men who have experienced suicidal thoughts or behaviours (n = 3, 25%).

### Quality ratings of quantitative articles based on the EPHPP protocol

Table 3 highlights areas where the ROB may be high for quantitative articles, based on the EPHPP protocol. Studies were generally not well controlled with four out of six articles being identified as weak on this criterion. Although studies generally performed well on the selection bias criterion, most quantitative studies that used a non-randomised, convenience sample obtained from prison inmate or psychiatric inpatient populations.

**Table 3:** Quality ratings based on the EPHPP protocol.

Study reference by first author	Selection bias	Study design	Confounder	Blinding	Measures	Attrition
Antypa [31]	1	2	3	2	1	2
Bryan [36]	1	2	3	2	2	1
Eidhin [38]	2	2	3	2	1	1
Humber [41]	3	3	2	2	1	1
Lekka [43]	2	2	1	2	3	1
Parker [32]	1	2	3	2	3	2

*Note*: According to the EPHPP protocol 1 = strong, 2 = moderate, 3 = weak fulfilment of the item criteria.

### Quality ratings of qualitative articles based on the CASP protocol

The results of the qualitative ROB ratings based on the CASP protocol are presented in Table 4, and indicate the quality of the qualitative research was generally high. The main source of bias was the lack of documented reflection on the relationship between the researcher and participants; the potential impacts of this on data collection and interpretation were not considered.

**Table 4:** Qualitative quality assessment led by the CASP assessment.

Study reference by first author	A) Aims	B) Methodology	C) Appropriateness	D) Recruitment	E) Data	F) Power	G) Ethics	H) Analysis	I) Clarity
Kiamanesh [42]	1	1	1	1	1	2	1	1	1
Peters [44]	1	1	1	1	1	1	1	2	1
Player [45]	1	1	1	1	1	3	2	1	1
Rasmussen [34]	3	1	1	1	1	3	1	1	1
Rasmussen [33]	1	1	1	1	1	3	1	1	1
Rivlin [46]	1	1	1	1	1	3	2	1	1

*Note*: According to the CASP protocol fulfilment of the item criteria is represented by 1 = yes, 2 = not clear, 3 = no. A) Was there a clear statement of the aims of the research? B) Is a qualitative methodology appropriate? C) Was the research design appropriate to address the aims of the research? D) Was the recruitment strategy appropriate to the aims of the research? E) Was data collected in a way that addressed the research issue? F) As the relationship between the researcher and participants been adequately considered? G) Have ethical issues been taken into consideration? H) Was data analysis sufficiently rigorous? I) Is there a clear statement of findings?

### Global ROB assessment

Following the global ROB assessment, two articles were excluded from inclusion in the final analysis for inadequate documentation of study survey materials and not establishing the reliability and validity the measures of suicide signs [32,43] (see Table 5). While the design of Humber et al [41] was initially rated as weak it was decided that, as this design was clearly documented, matched the hypotheses, and was able to assess suicide signs and suicidality in the near term, it was acceptable for inclusion. The remaining 10 articles were evaluated as adequate for inclusion.

**Table 5:** Article compliance and global ROB assessment rating for inclusion in final analysis.

Type of suicidal behaviour	First author	Original research article	Sample age 18>65	Assessed current suicidality	Male and female suicide signs reported separately	Identified suicide signs within 4 weeks of suicidality	Global ROB assessment	Included in study
Ideation								
	Eidhin[38]	✓	✓	✓	✓	✓	Strong	✓
	Humber [41]	✓	✓	✓	✓	✓	Strong	✓
	Lekka [43]	✓	✓	✓	✓	✓	Weak	✕
Attempt								
	Antypa [31]	✓	✓	✓	✓	✓	Strong	✓
	Bryan [36]	✓	✓	✓	✓	✓	Strong	✓
	Rivlin [46]	✓	✓	✓	✓	✓	Strong	✓
Death								
	Kiamanesh [42]	✓	✓	✓	✓	✓	Strong	✓
	Parker [32]	✓	✓	✓	✓	✓	Weak	✕
	Peters [44]	✓	✓	✓	✓	✓	Strong	✓
	Player [45]	✓	✓	✓	✓	✓	Strong	✓
	Rasmussen [33]	✓	✓	✓	✓	✓	Strong	✓
	Rasmussen [34]	✓	✓	✓	✓	✓	Strong	✓

### Male-specific suicide signs

#### Suicidal ideation

In quantitative studies, Humber et al [41] and Eidhin et al [38] identified anger, paucity of problem solutions, and social withdrawal which emerged in close proximity to suicidal ideation, but did not determine whether there was a causal relationship between these signs and the development of suicidal ideation (see Table 2). There were no qualitative articles identified in this review that investigated signs associated with suicidal ideation in men.

#### Suicide attempt

Two quantitative articles found that agitation and anger emerged in close proximity to suicide attempt but did not determine whether there was a causal relationship between these signs and suicide attempt [31,36](see Table 2).

Two qualitative studies both found that anger, calmness and direct statements of suicide intent were signs associated with suicide attempt in the near future [45,46]. Rivlin et al [46]also identified depression, feeling upset, relief, indirect or ambiguous references to taking their own life, and making arrangements and planning for death as commonly experienced signs associated with planning and preparing a suicide attempt. Player et al[45] reported aggression, apathy, hopelessness, and excessive risk taking were associated with suicide attempt.

#### Death by suicide

Anger [34,42], direct statements of suicidal intent [33,44], and paucity of problem solutions [33,42] were common signs associated with male deaths by suicide (see Table 2). Rasmussen et al [33] also found that in the days prior to death by suicide, many informants reported that conversations with the deceased were marked by an absence of negative mood, or the emergence of a more positive mood state, in addition to agitation, the perception of death as a problem solution, desperation and reaching out for support in desperation. Additionally, most studies identified that some level of statement of suicidal intent had occurred. The communication of suicidal intent sometimes occurred in ways that were not immediately obvious[33,44], such as talking about death as a problem solution or introducing actual or hypothetical suicide of someone else into conversation[33]. Informants reported that these suicide intentions were often difficult to recognise and were only identified in retrospect.

## Discussion

A systematic review of the literature was conducted to identify signs of suicidal ideation, attempt, and death by suicide in men. The findings of this review broadly reflect signs of suicide that have been identified through expert consensus[8]. However, the review also found that there is a paucity of research identifying male-specific signs of suicidal ideation, attempt and death. As suicide signs are frequently used as an educational tool to increase the suicide literacy of front-line community workers, telephone crisis operators, and the general public, future research needs to determine whether signs that are currently reported in the suicide literature are sufficient to identify suicidal men.

### Signs of suicidal ideation, attempt and death by suicide in men

The findings of the review identified a variety of suicide signs preceding suicidal ideation, suicide attempt and death by suicide in men. Signs of suicidal ideation in men were: social withdrawal, anger and reduced problem solving capacity. Signs of suicide attempt in men were: statements of suicidal intent, calmness, anger, apathy, hopelessness, risk-taking and appearing ‘at peace’. Signs preceding death by suicide in men were: desperation and frustration in the face of unsolvable problems, helplessness, worthlessness, statements of suicidal intent, and emergence of a positive mood state.

Patterns of suicide signs that differentiated suicidal ideation, attempt and death among men may be commented on while bearing in mind the limited number of articles identified by this review. Multiple studies identified anger as a suicide sign associated with suicide attempts among men [31,45,46]. A paucity of problem solutions was associated with suicidal ideation [38], but also associated with death by suicide among men[33,42]. Direct statements of suicidal intent were associated with both suicide attempt and death among men[33,44,46]. These findings are not definitive, and are based on studies with a variety of methodological approaches and levels of ROB. However, the pattern of findings reflect recommendations that any disclosure of suicidal ideation or intent must not be minimised or dismissed[47], particularly when this is paired with problem solving difficulties and experiences of intense negative emotion such as anger.

Although the signs found in this review reflect those identified by expert consensus, questions remain around the sensitivity and specificity of these signs to identify men experiencing suicidality. The broad range of emotional, behavioural and cognitive signs that, in constellation, may identify suicidal men have potential to prompt high rates of false positives. However, in suicide intervention, false positives that prompt a question or conversation about the possibility of current suicidality is not considered to be a negative outcome. “R U OK” is an Australian suicide prevention initiative that prompts community members to regularly ask each other about their current mood state and possibility of suicidality, even if there is only a suspicion that the individual might be suicidal. That said, the potential for false positives also highlights the need for to develop tools to assist the accurate identification of suicidal men. Research into suicidal presentation needs to be conducted with rigor and explore factors which may hinder helpers’ identification of people experiencing suicidality.

### Directions for future research

This review identified a wide range of designs and approaches to identify signs of suicidality in men, with few designs prospectively identifying signs that emerge immediately preceding suicidal ideation, attempt or death. This is evident through the number of studies in this review that used retrospective interviews with informants on suicide attempters and decedents to identify signs of suicide [33,34,42,45]. Recent research has found that there are consistent patterns in types of presenting features considered by helpers to indicate imminent suicide risk. Gould et al[48] found that telephone crisis supporters at a national telephone helpline identified four caller profiles that they considered to reflect imminent risk of suicide. Whether these profiles can accurately and reliably identify suicidality is tempered by research indicating that individuals’ identification of signs of suicide may be biased by assumptions about suicide and gender [47,49,50]. Combined with the findings of this review, this suggests that currently, most research reflects the interpretation, rather than expression of suicide signs, which may limit the utility of signs of suicide that are currently reported in literature for identifying suicidal men. In order to develop accurate education and training for family, friends, and front-line community workers, prospective research must be conducted to identify signs that indicate men are suicidal. Additionally, the issues around the sensitivity and specificity of suicide signs for accurately and efficiently identifying those who are suicidal highlights the need to investigate how signs of suicide are used by helpers and community-members to identify those in need of suicide intervention.

### Strengths and limitations

The strength of this review lies in the rigor and replicability of the PRISMA-P methodology, and the multiple checks and balances put in place to limit bias in data screening, extraction, and coding, including independent assessment and review. The articles included in this review were comprehensively assessed for risk of bias by multiple raters, to ensure the limitations of the included articles were thoroughly reviewed.

There are a few limitations to be noted. It is possible that the findings of this review have been impacted by publication bias. Due to the methodological heterogeneity of the study variables and the preclusion of meta-analysis, formal assessment of publication bias such as sensitivity analysis was unable to be conducted. Publication bias is particularly prominent in “gender-differences” research, as data supporting gender differences are more likely to be published than data in support of no difference [51].

Although many articles identified in this review contained male and female samples, few commented on gender differences beyond demographic information, which may reflect the tendency to not report null gender results. However, similarity in suicide signs between men and women should not be assumed, particularly due to the gendered nature of emotional communication [52]. As this review highlighted, the paucity of current research into signs of suicide means that future research should clearly examine the impact of gender on suicidal presentation because gender may be an influential factor in how suicidality is presented.

Finally, the small number of articles identified in the review prevented exploration of trends that may differentiate the signs associated specifically with ideation, attempt and death by suicide. To improve the sensitivity and specificity of suicide signs, future research should investigate whether certain signs are able to distinguish between suicide ideation, attempt and death by suicide.

## Conclusion

This review was conducted to identify signs of current suicidality in men and provide directions for future research. Without accurate information regarding signs of suicide in men, the lay public and clinicians may not be well-equipped to identify suicidal men in need for suicide intervention. Although the findings indicated general support for the signs identified by clinical consensus[8], this review found little research identifying signs of men experiencing current suicidality. As knowledge of suicide signs is an important feature of suicide literacy for both clinicians and the lay public[9], the paucity of this research is problematic. Several implications for future research were highlighted including the including the need for prospective research to identify signs of men experiencing suicidality. In order to improve the identification and response to those experiencing thoughts of suicide a nuanced approach, exploring the influence of gender, culture and other individuating factors on the expression of suicidality, must be taken.

## Supporting information

S1 Table Summary of findings and suicide sign label. (DOCX)

S2 Table Suicide sign label and description. (DOCX)

S3 Table PRISMA Checklist. (DOCX)
