# Supplementary material for: Signs of current suicidality in men: A systematic review
Source: PLoS One. 2017 Mar 29;12(3):e0174675. doi: 10.1371/journal.pone.0174675 (PMC5371342; doi:10.1371/journal.pone.0174675)
Supplement: S1 Table — (DOCX) [file pone.0174675.s001.docx]

**S1 Table. Summary of findings and suicide sign labels.**

| **First author** | **Summary of findings** | **Text example** | **Suicide sign code** |
| --- | --- | --- | --- |
| Antypa (2013) [31] | - STAXI subscales differentiated male and female suicide attempters from controls | - N/A | - Anger |
| Bryan(2014) [36] | - Agitation severity differentiated male patients with a history of suicide attempts from male patients who were thinking about suicide, but had not yet made an attempt. | - N/A | - Agitation |
| Eidhin (2002) [38] | - - The current ideator and past suicide attempt group had significantly higher mean privacy preference scores than the control group, and social stimulation preference was significantly higher in the control group compared to current ideator and past suicide attempter groups. - The control group generated significantly more active problem solutions than the other two groups. The current ideator group generated significantly more passive problem solutions than the past attempt group. | - N/A | - Paucity of problem solutions - Social withdrawal |
| Humber (2013) [41] | - Being angry was concurrently associated with self-harm ideation 'I want to hurt myself'. High externalised anger predicted concurrent self-harm ideation. Items positively skewed to assess suicidal thoughts e.g., ‘I want to live’, were not associated with anger experience or expression. There was a temporal (delayed) relationship between high internalised anger and thoughts of wanting to live. | - N/A | - Anger |
| Kiamanesh (2014) [42] | - Some informants identified that the decedent’s façade cracked in the last few weeks of their lives, losing the belief in their ability to live up to their own expectations. The strain was indicated by behaviour changes such as insomnia; emotional changes such as loneliness; cognitive changes such as an inability to problem solve. | - *“All the informants considered the adversities prior to the suicides (financial crisis, relationship break-up, trouble at work place, etc.) to have been insignificant on their own. However, in hindsight, the informants noted that the deceased person must have perceived these challenges as irreversible defeats and as failures of the whole self, and eventually triggering the suicide” (p. 319)* - *‘‘In the four weeks leading up to the suicide he seemed like a different person. He wasn’t sleeping much, cried almost every day and felt like a failure… lost control, and lost himself in the process.’’(p.319)* - *“In two deaths, a short time before they ended their life, the deceased persons had expressed feelings of loneliness and that they ‘‘could not take it any longer.’’ (p.320)* - *“…the deceased person seemed ‘‘stuck in a circle with no way out.’’ This phenomenon seemed to have been the case for all these men.” (p.320)* | - Irreversible defeat - Lack of sleep - Loneliness - Paucity of problem solutions |
| Lekka (2006) [43] | - Feelings proceeding thoughts about suicide were summarised with descriptive statistics. The most common feelings causing suicidal ideation among male prisoners was anger, guilt, hopelessness, wish to die and wish for change. |  | Excluded from study |
| Parker (2002) [32] | - Approximately 70 per cent of Aboriginal males and “other” females were recognized by others as having one or more features of behaviour which may have been characteristic of mental illness prior to their deaths. Depressed mood was the most commonly observed sign in both female populations. For both the Aboriginal and “other” male populations, the most common signs were behaviour that was out of character, aggression, depressed mood and anxiety or agitation. |  | Excluded from study |
| Peters (2013) [44] | - Purposeful indications of intent to die included direct statements of suicidal intent. | - *“All but one of the decedents had had at least one prior attempt to take their life by suicide or had openly verbalised their intent to end their life” (p.311)* | - Direct statements of suicidal intent |
| Player (2015) [45] | - Disrupted mood often included an increase in anger, aggression and violence, social isolation, as well as increased suicidal thinking. Participants reported that acute suicidal thinking was associated with excessive risk-taking, overt statements of intent, hopelessness, apathy or appearing ‘at peace’. | - *“Participants reported that acute suicidal thinking was associated with excessive risk-taking, overt statements of intent, hopelessness, apathy or appearing ‘at peace’.” (p. 13)* - *“Several friends and family members also observed that depressed and suicidal males often expressed anger or aggression instead of, or to avoid feelings of sadness, anxiety or stress. However, angry, aggressive or defensive behaviour was also likely to strengthen social isolation by increasing risk of interpersonal conflict, reinforcing a sense of disconnection, and lowering motivation for family and friends to offer help.” (p. 13)* | - Aggression - Anger - Apathy - Calm - Direct statements of suicidal intent - Excessive risk taking - Hopelessness - Social isolation |
| Rasmussen (2014) [33] | - Several warning signs of suicide in the weeks prior to death were observed including; uncharacteristic restlessness, deceased was stuck in a problem situation with no possible way out, unsuccessful requests for support and emotional safety, desperation, through talk or action threatened to take their own life, direct or indirect discussions about death as a ‘place to go’, emotional calm and cheerfulness, introduced actual or hypothetical suicide of somebody else into conversation. | - *“In the last weeks of life, he was restless and insecure, and repeatedly complained that he felt his achievements at work were not good enough any longer, although they were outstanding….Similarly, in another case, referring to her experiences of her son in the last week before he killed himself, a mother said, “then he was like, so restless and seemed . . . like we had never seen the boy before”. In these two examples, the informants are referring to how they, in their last conversations had experienced signs of desperation, in particular related to an uncharacteristic restlessness.”(p.8)* - *“… Many male informants pointed to how the deceased in their last conversations had been more cheerful and social than usual, as well as being almost desperately focused on planning things together.” (p. 10)* - *“…talk (direct or indirect) of death as a place to go was experienced as a sign of danger of suicide in near future and/or of suicide planning related to a need to be free of demands” (p. 10)* - *“Thus, desperation as a warning sign of suicide was related to the experiences of the deceased in the last days of life, too constrained to handle difficulties in love and work, were entrapped in a problem situation and unable to move on in life.”(p.8)* - *“…many male informants in different cases pointed to how they, in their last conversation(s), had noticed signs of a kind of cheerfulness which, in retrospect, was interpreted as relating to death being considered a release.” (p.10)* - *“In one case, after the funeral of a friend, several informants described how they had noticed that the deceased had become increasingly occupied with wishes for his own funeral in the months before he killed himself” (p.9)* - *In different cases, friends pointed to how the deceased had unexpectedly introduced death and/or of an actual or hypothetical suicide of somebody else into their conversations in the weeks prior to the suicide. The informants, in retrospect, interpreted this as warning signs of suicide planning. (p.10)* - *Thus, desperation as a warning sign of suicide was related to the experiences of the deceased in the last days of life, too constrained to handle difficulties in love and work, were entrapped in a problem situation and unable to move on in life.* - *“… most of the deceased had unsuccessfully approached their ex-girlfriends and/or mothers with a personal request in the last day(s) of life. According to these ex-girlfriends, the clue of something being wrong was related to how the personal request had been “misplaced in time and place” (p.7)* - *“…Suggestive of a kind of relief, after having made the final decision that would put an end to all demands” (p.10)* | - Agitation - Cheerfulness - Death as a problem solution - Desperation - Direct statements of suicidal intent - Emergence of positive change of mood - Indirect or ambiguous references to taking their own life - Paucity of problem solutions - Reaching out for support in desperation - Relief |
| Rasmussen (2014) [34] | - Informants identified the decedents exhibited signs of shame, helplessness, and anger prior to death. | - *“In most cases, several informants linked episodes of anger outbursts in the weeks, months, or last years prior to the suicides to their understanding of being “trapped in anger.”… These informants shared a notion of how feelings of shame, helplessness and anger had become entrapped in the deceased.”(p.554)* | - Anger - Helplessness - Shame |
| Rivlin (2013) [46] | - Three of the most common reported changes prior to suicide were depression, and feeling upset, or angry. Several participants also spoke of their state of mind in positive terms including relief or feeling pleased to have made the decision to end their own life, and a sense of calm or peacefulness. Impulsivity was described by many participants. - Participants reported their suicidal intent in a variety of ways including; explicitly or ambiguously telling someone, writing a note, or made arrangements in anticipation of death. | - *“The prisoners were asked to explain how they felt once they had decided to attempt suicide and whilst they were planning and preparing the act. The three most common feelings were depression (13/55, 24%), and feeling upset (12/55, 22%) or angry (8/55, 15%).” (p. 313)* - *“A sense of calm or peacefulness, or happiness, was also described by some prisoners: I was feeling dead calm then. Really calm. Calm like I’m speaking to you. That’s how calm I was. (Case 23)” (p. 313)* - *“Approximately one-third of prisoners had explicitly told someone (for example, a family member or psychiatrist) of their wish to die. Two (3%) prisoners said that they had made ambiguous references to family that they might take their own lives.” (p. 314)* - *“…some prisoners were relieved or pleased to have made the decision to end their lives: A bit of relief to be honest. It wasn’t ‘oh bloody hell this is going to hurt’, or ‘oh no I’m going to die’. I just felt relief . . . a way out. (Case 16)” (p. 313)* - *“Twenty-one (35%) prisoners wrote a suicide note and seven (12%) thought about it but never did it. Seven (12%) prisoners made a definite arrangement in anticipation of death, for instance giving instructions to a solicitor or making child-care arrangements.” (p. 314)* | - Anger - Calm - Depression - Direct statements of suicidal intent - Feeling upset - Indirect or ambiguous references to taking their own life - Made arrangements for death - Planned suicide attempt - Relief - Wrote a suicide note |
