# Supplementary material for: Signs of current suicidality in men: A systematic review
Source: PLoS One. 2017 Mar 29;12(3):e0174675. doi: 10.1371/journal.pone.0174675 (PMC5371342; doi:10.1371/journal.pone.0174675)
Supplement: S2 Table — (DOCX) [file pone.0174675.s002.docx]

**S2 Table**. **Suicide sign label and description.**

| **Suicide sign label** | **Description** | **Label synonyms identified across articles** | **Reference** |
| --- | --- | --- | --- |
| Social withdrawal | A change in behaviour in which a person withdraws from situations that involve social interaction. | Decreased preference social stimulation, increased privacy preference. | Eidhin (38) |
| Anger | Anger is an emotion with a wide range of intensity, from mild irritation to frustration and rage, with a range of cognitive and behavioural features. | Angry affect, internalised anger, externalised anger, state anger, expressed anger | Humber (41); Antypa (31); Player (45); Rivlin (46); Rasmussen (34); |
| Paucity of problem solutions | The inability to develop active solution to problem situations. | Increased passive problem solving, decreased active problem solving; stuck in a problem with no possible way out; trapped in an unsolvable situation ; Feeling irreversible defeat | Eidhin (38); Rasmussen (33); Kiamanesh (42) |
| Agitation | An acute state of psychological and physiological over-arousal characterised by behavioural restlessness and emotional unrest. | Agitation, emotional urgency, uncharacteristic restlessness; | Bryan (36); Rasmussen (33) |
| Upset | A feeling of unhappiness or sadness. |  | Rivlin (46) |
| Apathy | A state of indifference; a lack of feeling, emotion, interest or concern for self or others. |  | Player (45) |
| Calm | A feeling of tranquillity and peacefulness, absent of strong emotions. | A sense of calm, peacefulness, happiness, dead calm ; appearing ‘at peace’ | Rivlin (46); Player (45) |
| Cheerful | Feeling or behaving in a way expressing good spirits and happiness. | Elated; happiness | Rassmussen (33) |
| Relief | A feeling of ease or relaxation following the removal of pain and distress. | Pleased to have made decision to end life. | Rassmussen (33) |
| Absence of negative mood – emergence of a more positive state | A distinct change from a negative mood state to the emergence of a more positive mood state. |  | Rasmussen (33) |
| Direct statements of suicidal intent | A person explicitly told someone of their wish/intent to die. | Overt statements of intent; through talk or action threatened to take their own life | Rivlin (46); Player (45); Peters (44); Rasmussen (33) |
| Indirect or ambiguous references to taking their own life | A person made an ambiguous reference to someone that they might take their own lives. | Introduced an actual or hypothetical suicide of someone else into conversation | Rivlin (46); Rasmussen (33); Kiamanesh (42) |
| Death as a problem solution | A person discussed death as a problem solution |  | Rasmussen (33) |
| Wrote a suicide note | A person wrote a suicide note. |  | Rivlin (46) |
| Made arrangements for death | A person made a definite arrangement in anticipation for death, for instance, giving instructions to a solicitor or making childcare arrangements. |  | Rivlin (46) |
| Hopelessness | A feeling that there is no possibility for improvement, change or success in the future. |  | Player (45) |
| Aggression | Feelings of anger or antipathy which result in hostile or violent behaviour. | Expressed aggression | Player (45) |
| Excessive risk taking | Behaving in a way that needlessly incurs danger or risk. |  | Player (45) |
| Desperation | Out-of-character behaviour driven by desperation. | Reaching out for support in desperation | Rasmussen (33) |
| Shame | A painful feeling of distress caused by being aware of a persons’ own wrong or foolish behaviour. |  | Rasmussen (34); Rasmussen (33) |
| Helplessness | Being unable to help oneself, powerlessness to change the situation. |  | Rasmussen (34) |
| Lack of sleep | A condition of not having enough sleep. |  | Kiamanesh (42) |
| Loneliness | The perception of being alone and isolated from other people. |  | Kiamanesh (42) |
| Planning suicide attempt | Developing a plan to take ones’ own life. | Precautions against discovery, barricading door and furniture | Rivlin (46) |
